# Tibial Stem Extension versus Standard Configuration in Total Knee Arthroplasty: A Biomechanical Assessment According to Bone Properties

**DOI:** 10.3390/medicina58050634

**Published:** 2022-05-02

**Authors:** Alexandru Cristian Filip, Stefan Alexandru Cuculici, Stefan Cristea, Viviana Filip, Alexis Daniel Negrea, Simona Mihai, Cosmin Marian Pantu

**Affiliations:** 1 Radiology and Medical Imaging Department, ‘Dr. Carol Davila’ Central University Emergency Military Hospital, 010825 Bucharest, Romania; alexandru.filip95@yahoo.com; 2 Department 8—Radiology, ‘Carol Davila’ University of Medicine and Pharmacy, 050474 Bucharest, Romania; 3 Department of Orthopedic Surgery, Ilfov County Emergency Clinical Hospital, 022104 Bucharest, Romania; 4 Department of Orthopedic Surgery and Trauma, ‘Sf. Pantelimon’ Emergency Clinical Hospital, 021659 Bucharest, Romania; drstefancristea@yahoo.com; 5 Department 14—Orthopedics, ‘Carol Davila’ University of Medicine and Pharmacy, 050474 Bucharest, Romania; 6 Mechanical Department, Doctoral School, ‘Valahia’ University, 130004 Targoviste, Romania; v_filip@yahoo.com; 7 Mechanical Department, Materials and Mechanical Faculty, ‘Valahia’ University, 130004 Targoviste, Romania; alexis.negrea@yahoo.com; 8 Mechanical Department, Institute of Multidisciplinary Research for Science and Technology, ‘Valahia’ University, 130004 Targoviste, Romania; mihai.simona@yahoo.com; 9 Department 2—Morphological Sciences—Anatomy, ‘Carol Davila’ University of Medicine and Pharmacy, 050474 Bucharest, Romania; pantucosmin@gmail.com

**Keywords:** total knee arthroplasty, primary total knee arthroplasty, implant, prosthesis, tibial stem extension, osteoarthritis, finite element analysis

## Abstract

*Background and Objectives*: This study’s purpose was to examine the benefit of using a tibial extension in the primary operation of total knee arthroplasty (TKA). This is important because it is not a common practice to use the extension in a primary TKA, a standard configuration offering sufficient stability and good long-term survivorship. The following question arises: which situation requires the use of a standard configuration implant (without a stem) and which situation requires using the extension? *Materials and Methods*: The opportunity to use the tibial extension in the primary TKA was analyzed in correlation to the tibial bone structural properties. Using finite elements (FEs), the virtual model of the tibial bone was connected to that of the prosthetic implant, with and without a stem, and its behavior was analyzed during static and dynamic stresses, both in the situation in which the bone had normal physical properties, as well as in the case in which the bone had diminished physical properties. *Results:* The maximum stress and displacement values in the static compression regime show that adding a stem is only beneficial to structurally altered bone. Compression fatigue was reduced to almost half in the case of altered bone when adding a stem. Dynamic compression showed slightly better results with the tibial stem in both healthy and degraded bone. *Conclusions*: It was concluded that, if the bone is healthy and has good structural properties, it is not necessary to use the tibial extension in the primary operation; and if the bone has diminished physical properties, it is necessary to use the tibial extension at the primary operation, enhancing the stability, fixation, and implant lifespan.

## 1. Introduction

**Figure 1:**
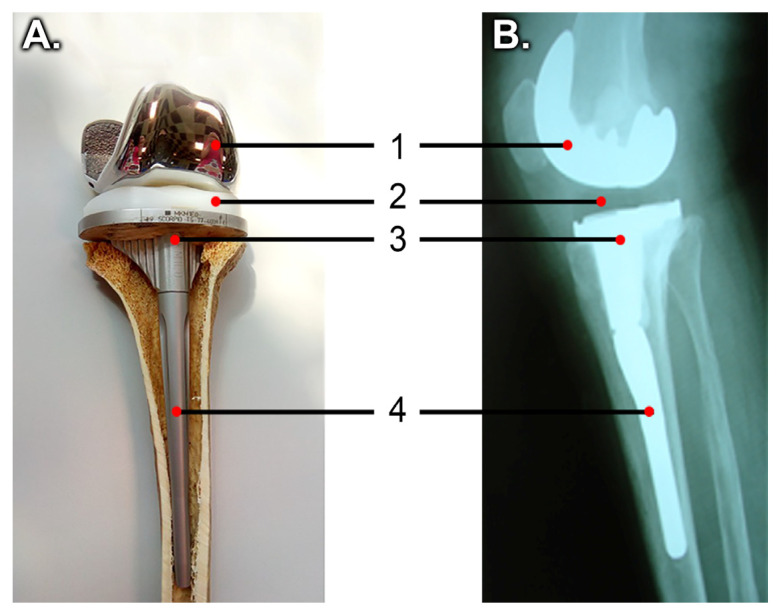
Knee arthroplasty: (**A**) Tibial bone with prosthesis, laboratory model, (**B**) Postoperative radiograph ((1) femoral metal implant, (2) polyethylene spacer, (3) tibial metal implant, and (4) tibial stem extension).

Total knee arthroplasty (TKA) is the surgical intervention in which cartilage and damaged underlying bone of the knee are excised by the surgeon and replaced with a prosthesis, consisting of a (1) femoral metal component, (2) polyethylene spacer, and (3) tibial metal component (Figure 1). This implant restores the mobility of the knee joint [1]. In some cases, to improve the fixation and stability of the implant, at the distal end of the tibial metal component, an extension with a cylindrical shape, called tibial stem (Figure 1), is added [2].

The most common problems that appear after total knee replacement surgery are wear of the polyethylene insert and loosening of the femoral and tibial components, or even their breakage.

The role of a tibial stem in the primary TKA is to enhance the stability of the implant in complicated cases [3,4,5,6,7]; this is achieved by reducing micromotions at the bone–implant interface and, as a result, the risk of aseptic loosening, which is one of the main causes of failure [8,9]. Tibial extensions have different lengths and widths and can be fixed in different ways, either by full cementation, only proximal cementation, or press-fit, without cement. 

Medical practice reveals the following situations of a complicated primary TKA where a stem can be effective: when the bones have altered physical properties created by various diseases (osteoporosis, osteoarthritis, and rheumatoid arthritis), when the patients have extreme varus–valgus deformities [10,11,12,13], when there is a pre-existing proximal tibial defect, when there is a previously performed corrective osteotomy, when there is a large proximal tibial defect or necrosis, when the bones have malunited fractures, and in the situation when the patients’ particularities such as age or lifestyle overexert the implant [14,15]. Moreover, there is the case of some unexpected intraoperative events that may require additional implant fixation [16,17,18].

It is crucial for the survivorship of the implant to achieve a very good alignment of the femur, tibia, and patellar components of the implant during the total knee replacement surgery [19]. This can reduce both the mechanical stress forces on the bearing surfaces and the shear forces at the bone–cement and bone–prosthesis levels. Moreover, a good alignment helps to balance the forces transferred to the soft tissues, which are essential for proper joint mobility.

The presence and configuration of the tibial extension (size and mode of fixation) [20,21,22,23] can determine a phenomenon known as stress shielding, leading to bone loss in the areas where it is higher. This can further weaken the implant’s fixation, requiring the implantation of a new prosthesis.

The need for reoperation, or revision surgery, is about 14–15%, and almost 44% of them may require more than two supplementary interventions [24,25,26,27]. If a tibial extension is used during the primary knee arthroplasty, then, during the revision surgery, it will have to be removed and replaced with a new extension. A very large bone tissue resection might be necessary in the case of a revision when removing the implant, leaving behind a low bone stock and technical problems for fixing the revision implant [28]. This explains the excellent long-term results generally obtained when replacing the natural knee with the orthopedic implant in the standard configuration (without tibial extension) and makes this design the best option in the primary TKA [29].

The main pathology where a TKA is needed is knee osteoarthritis. There are two forms of osteoarthritis that have different causes, but they manifest with the same symptoms.

Situation 1: The natural knee joint cartilage was destroyed as a result of a triggering factor. These triggers can be trauma, obesity, inactivity, inflammation, and genetics. This is called secondary osteoarthritis. In this case, the physical properties of the tibial bone are not always affected, behaving similarly to a healthy bone, during the initial stages of the pathology.

Situation 2: The natural knee joint has been destroyed as a result of normal aging. Everyone experiences a form of this pathology after a certain age, with some cases being more severe and others not. This is called primary osteoarthritis, where the bone is usually affected, and its properties are diminished compared to those of healthy bone.

The behavior over time differs in the two situations, and the medical decision may be different regarding the choice of the type of implant.

The question arises as to whether or not, according to the quality of the tibial bone, the tibial extension is necessary.

We hypothesized that primary TKA patients with pre-existing conditions and a modified bone structure would benefit from adding a modular stem to the tibial implant.

The finite element (FE) method [30,31] has been used extensively in orthopedic biomechanics for evaluating the behavior of bone tissue and prosthetic implants. This method determines the tensile forces present in the bone tissue and establishes the connection with biological processes, such as bone remodeling [32,33,34], by creating a mathematical model that simulates this behavior.

In order to help the decisional medical act, in the present work, a study was performed by using FE analysis. Therefore, by testing the bone–implant assembly’s behavior, both in the version without tibial extension and also with tibial extension, both for Situation 1 (healthy bone, with good physical properties) and for Situation 2 (bone with diminished physical properties), the conclusions are presented.

## 2. Materials and Methods

The digital model of the knee implant and tibial bone was made, and the bone–implant virtual assembly was analyzed, both from a kinematic point of view and from a mechanical strength point of view, by using finite element analysis techniques.

**Figure 2:**
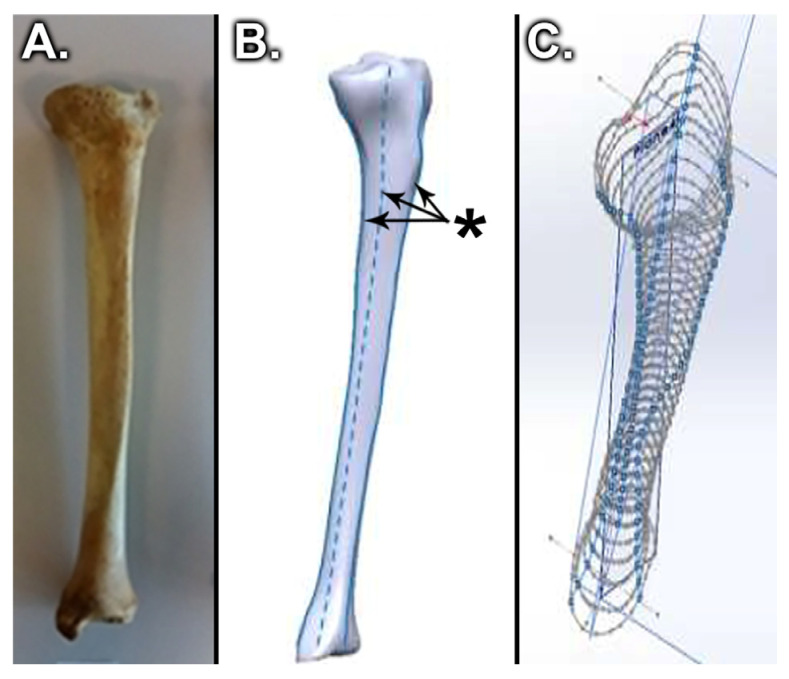
Conversion of a real tibial bone to a virtual model [35]: (**A**) Tibial bone, (**B**) Scanned model of the tibial bone with guidelines for creating the virtual model, (*) – marks on scanned model and used as guidelines for creating the virtual model, (**C**) Virtual tibial bone model during processing.

**Figure 3:**
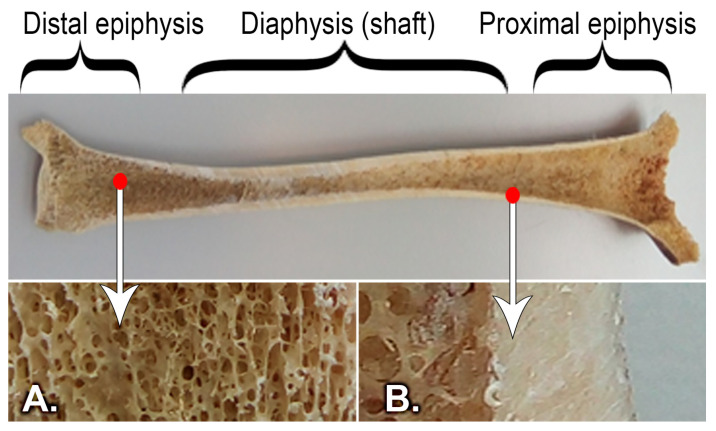
Structure of the tibial bone: (**A**) Cancellous bone, (**B**) Cortical bone.

In the process of creating the virtual tibia model (Figure 2C), a digital model was used as a reference model (Figure 2B), and it was obtained by scanning a real bone (Figure 2A) [35,36]. The virtual model that we obtained had distinct material properties assigned to it, determined by the level of the bone and considering the following specifications (Figure 3): the tibia (as most long bones) is formed by a shell of compact bone substance, covering a cancellous bone substance at the two proximal and distal parts, and having the medullary canal in the center. These types of bony substances behave differently because they have different bone mineral densities and properties [37,38,39].

The mechanical properties of healthy and unhealthy bones were chosen as average values extracted from the existing specialty literature [40,41]. Three virtual materials were modeled in the Solidworks (Dassault Systèmes Solidworks Corp., Vélizy, France) simulation: cortical bone, healthy cancellous bone, and unhealthy cancellous bone, to which the values present in Table 1 were attributed.

**Figure 4:**
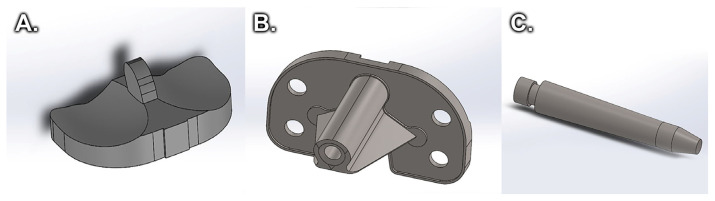
Tibial prosthesis components: (**A**) Polyethylene insert, (**B**) Tibial component, (**C**) Tibial stem.

After constructing the tibial bone model, the virtual model of the knee prosthesis was made, consisting of a polyethylene spacer (Figure 4A), tibial component (Figure 4B), and tibial extension (Figure 4C) [2].

The mechanical properties of the implant and cement components were chosen from their manufacturers’ catalogs. The implant components were modeled in Solidworks, and virtual materials were assigned the characteristics from Table 2.

Four study models were designed: completely healthy bone (cortical and cancellous); damaged bone (good cortical and unhealthy cancellous); and 2 situations, one with and one without the presence of a tibial intramedullary stem.

**Figure 5:**
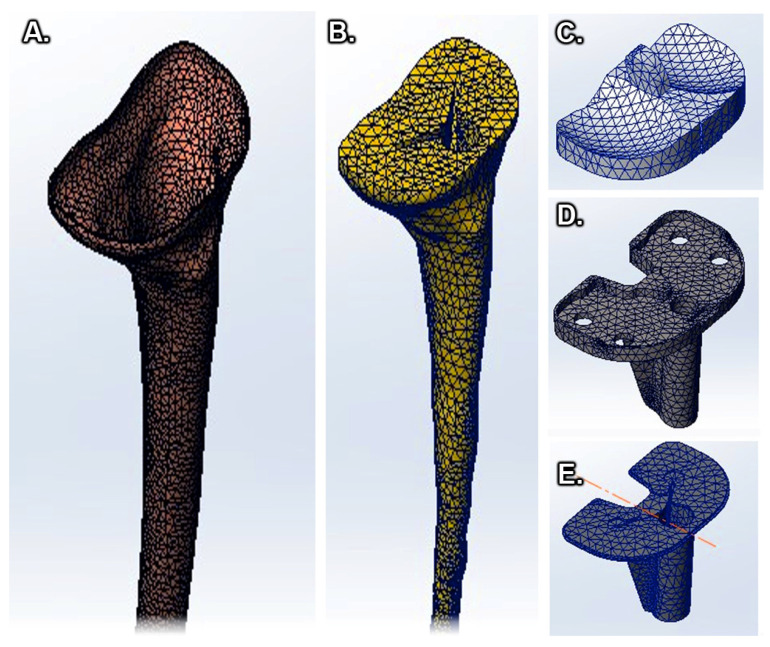
Tibial bone–implant model: (**A**) cortical bone, (**B**) cancellous bone, (**C**) polyethylene insert, (**D**) tibial tray, and (**E**) cement mantle.

All four study models consist of an outer layer of cortical bone with an increasing thickness from 0.8 mm in the proximal and distal epiphysis to 4 mm in the diaphysis. (Figure 5). The two numerical values were obtained by direct measurement of cadaveric bones. The model also contains a part of cancellous or spongy bone, healthy or unhealthy, positioned in the proximal, diaphyseal, and distal epiphysis; the tibial tray with and without the tibial extension; a thin layer of cement; and the polyethylene insert.

**Figure 6:**
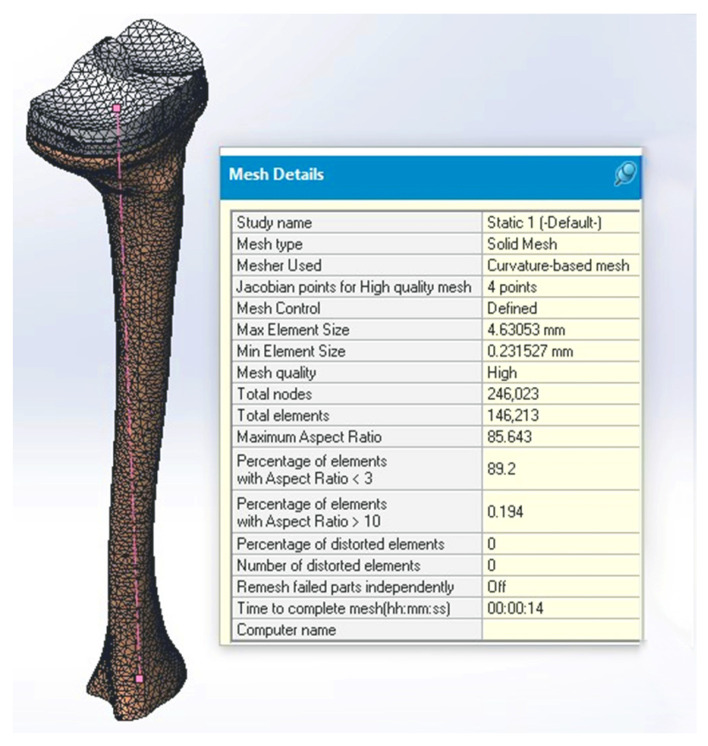
Tibial bone–implant model.

To simulate the in vivo behavior of the implant, the tibial bone–knee implant assembly was created (Figure 6).

The same conditions and parameters were applied on all four models to simulate the behavior of dynamic stress and fatigue. We pursued certain stages in the FE study.

The first stage was establishing contact between the components of the model which we chose to be bonded. As for connecting the tibia to the external environment, we fixed the tibia at the distal epiphyseal part.

**Figure 7:**
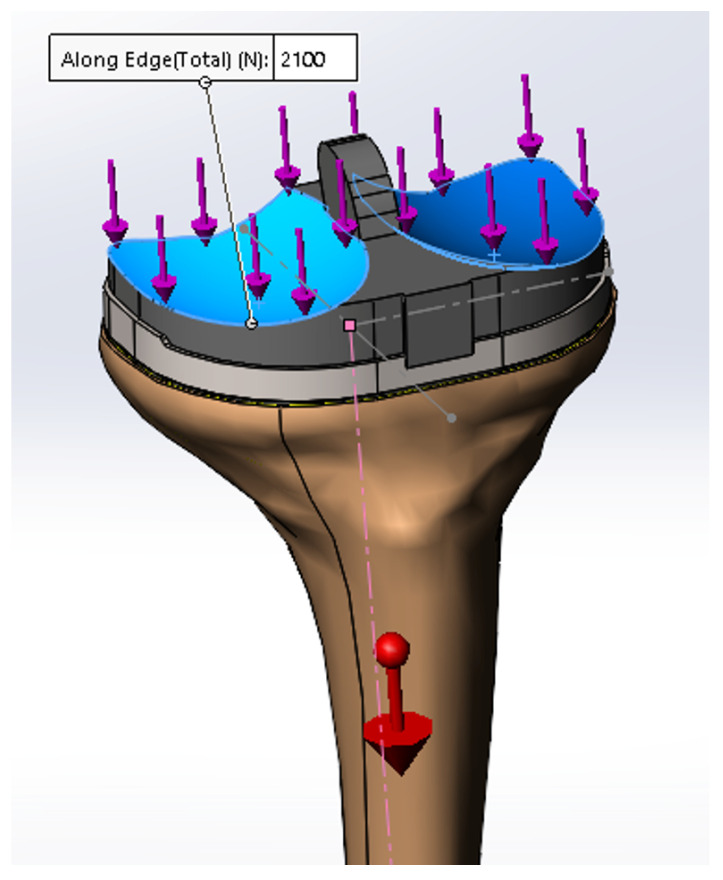
Force evenly distributed over the two contact surfaces.

The implementation of external forces was made based on type, direction of action, their nature, and intensity (Figure 7). Since the main stress the implant is exposed to is compression, a compressive force of 2100 N was applied in the direction of the longitudinal axis of the tibial bone. The value of a 2100 N force is used throughout the literature for testing knee arthroplasties, and it is equal to the average weight of a patient multiplied by three. The behavior at static compression stress was used to appreciate the behavior in the orthostatic position, the fatigue stress to appreciate the implant’s life, and the dynamic compression regime was used to study the behavior while walking and running.

### 2.1. Static Compression Behavior

We followed the distribution of tension (stress), general displacements, *z*-axis displacements, contact pressure, and the safety coefficient, both for healthy bone and for bone with diminished physical properties, for both the case of the implant without tibial extension and for the case with tibial extension.

### 2.2. Compression Fatigue

The material that is subject to the cyclic variation of the mechanical loads can be damaged over time, even if the stresses it is subject to are lower than the flow limit. A microcrack can appear, it can propagate, and, finally, the material can break. We determined the number of stress cycles at which the fatigue occurred in several representative points of the tibial bone–knee implant.

### 2.3. Stress in Linear Dynamic Regime

The loads used in the linear dynamic analysis can be predictive or non-predictive, and in the second case, they can be statistically defined. The reaction of the analyzed system can be important, so taking into account its inertia and absorbing forces is essential. The following formula was utilized:

(1) $$[M]\;\left\{\ddot{u}\left(t\right)\right\}+[C]\;\left\{\dot{u}\left(t\right)\right\}+[K]\;\{u(t)\}=\{f(t)\}$$

where [C] = damping matrix, [K] = stiffness matrix, [M] = mass matrix, {u(t)} = time varying displacement vector, $\left\{\ddot{u}\left(t\right)\right\}\;$= time varying acceleration vector, $\left\{\dot{u}\left(t\right)\right\}\;$= time varying velocity vector, and {f(t)} = time varying load vector [42].

The assembly was analyzed with finite elements for linear dynamic regime, as the force was increased linearly from 0 to 2100 N within one second. The analysis was performed both for healthy bone and for damaged bone.

## 3. Results

Table 3 summarizes the maximum stress and displacement values in the static compression regime, without and with tibial stem extension, in the case of both healthy bone and damaged bone.

The stress values are collected at the tibial-bone level, and the displacement values are from a point of the tibial metal component, in both the case of the tibial bone without stem and the case with stem.

As can be seen in Table 3, unhealthy bone has a lower stiffness than healthy bone, meaning that the maximum stresses in the tibia that occur in unhealthy bone are lower than those that occur in healthy bone for the same loading conditions, both in the stemless version and in the stemmed version.

Analyzing the values in Table 3, it is found that, when the bone has good physical properties (healthy bone), the maximum stresses have comparable values (18.04 MPa in the version without tibial extension, and 21.2 MPa in the version with tibial extension), and, also, the maximum displacements have comparable values (0.1148 mm in the variant without tibial extension, and 0.1192 mm in the variant with tibial extension), meaning that adding the tibial extension does not bring a decrease of the maximum values of tensions and displacements, signifying that an implant in standard configuration without tibial extension is sufficient.

In contrast, in the case of structurally damaged bone, the values mentioned in Table 3 show that the introduction of the tibial stem extension reduces to half the maximum values of stresses (from 15.85 MPa in the version without tibial extension to 6.69 MPa in the version with tibial extension) and displacements (from 0.1047 mm in the version without tibial extension to 0.0645 mm in the version with tibial extension), thus signifying that, when the physical properties and mineral density of the bone are diminished, the introduction of the tibial extension is necessary from the primary replacement surgery.

Regarding the fatigue stress on compression, it is found that, both in the version with and in the version without tibial extension, the area where the fatigue occurs most likely is the distal part of the tibia, toward the ankle.

In Table 4, we compare the number of cycles in which the phenomenon of fatigue appeared in two different locations, Point 1 and 2, from the distal part of the tibia, which we considered representative.

As shown in Table 4, in the case of structurally healthy bone, adding the tibial stem does not increase the number of stress cycles to fatigue in either representative points, so it does not increase the resistance to fatigue, while, in the case of degraded bone, adding the tibial extension leads to the doubling of the fatigue resistance of the bone–implant assembly in both representative points (4,168,008 cycles compared to 1,668,583 cycles, respectively; 5,834,292 cycles compared to 2,501,725 cycles, respectively).

Regarding the compression stress in linear dynamic regime for healthy bone and for damaged bone, the maximum values of stress and displacement are synthesized in Table 5, as well as the values of stresses and displacements in two representative points: Point 1 (below the tibial plateaus) and Point 2 (at the distal tip of the implant).

A fatigue analysis was performed based on the initial load (2100 N, Figure 7) and the results obtained (Table 3). The fatigue failure is given by the number of repeated stress cycles at which the rupture occurs. Admitting that a person performs 2000 cycles/day for × 365 days, meaning 730,000 cycles per year, we subjected the model to a number of 10,000,000 cycles that we estimated the patient with the implant to perform in approximately 14 years.

Looking at the values in Table 5, we conclude that the maximum stresses in the version with tibial extension have values close to those in the version without tibial extension, both in the case of healthy bone and in the case of damaged bone. The tensions measured in Points 1 and 2 are slightly lower in the version with tibial extension, compared to those in the version without tibial extension. All recorded values are below the permissible material limit.

The maximum values of the general displacement, as well as those of the *z*-axis displacement, in the case with tibial extension, are close to those in the case without tibial extension, both in the alternative of healthy bone and in the one of damaged bone. The displacements measured in Points 1 and 2 have slightly lower values in the tibial stem variant, both in the case of healthy bone and in the case of degraded bone.

## 4. Discussion

The aim of this study was to compare the behavior of a modular tibial implant with a standard configuration, which is mostly used in a primary non-complicated TKA to a stemmed configuration usually used in a revision TKA, but also appropriate in some select complicated primary TKAs. Our hypothesis that adding a tibial stem would increase the stability of an unhealthy bone was proven to be correct. The concern for increasing the life of the implant is a current scientific interest for researchers around the world.

Studies in the literature deal extensively with extension intramedullary stems in the revision TKA, but not as extensively in the primary TKA [20,21,22,24,25,43,44]. Medical practice shows that, if, in a total knee arthroplasty, a tibial stem extension is used, after a period of time, the phenomenon of stress shielding or bone loss occurs due to the abnormal distribution of loads to the bone, followed by the wear and tear of the implant and ultimate failure of the arthroplasty. The longer the stem, the higher the stress shielding, which is not desirable in a primary TKA, and an unfortunate necessity in a revision TKA [43].

We decided to exclude the femoral part of the implant from the biomechanical study, since it has been proven that, even a stemmed modular femoral implant minimally impacts the failure risk of the proximal tibia [31].

Some differences exist between this study and the findings of Frehill et al. (2010), who studied the setting of a revision TKA with a tibial defect, and found that, contrary to the commonly accepted belief, a stemmed implant can increase the stress in the implant–bone contact area [45].

However, in some FE element previous studies, a reduction of tibial strain ranging from 20% to 60% has been achieved with the use of stems, and also according to the structural stiffness of the bone [46,47].

Therefore, the use of a knee prosthesis with tibial stem is not necessary in most cases; overall, a standard configuration is the desirable choice. However, there are situations in which it is appropriate to use the tibial extension. One of these situations is when the bone is affected by various diseases, so it has lower resistance properties and a lower mineral density than healthy bone. Medical practice shows that, in this situation, using the stem extension is the proper solution.

The limitations of the study were the accuracy of the finite elements model obtained by scanning a tibia model. A very large computational power is necessary to create an accurate model, and sometimes this can be an obstacle. An alternate modeling technique proposed by the research community is using a CT scan of either a patient or a human cadaveric specimen, the quality of the reconstruction model being negligibly higher than that of an optical 3D model [48,49]. Mahmoudi et al. (2018, 2020) used the CT scan data to create the inner surface of the bone model [50]. In our study, this inner surface is modified, as in real TKA surgery itself, to accommodate the shape and size of the tibial modular component. Moreover, some studies showed that the results can be more precise if, in parallel with the cancellous bone, the mechanical properties assigned to the cortical bone also have lower values, thus simulating the human anatomy better [51]. Another limitation is the absence of muscle and ligament strains that act in the knee joint and which may cause asymmetry in the forces applied in our finite elements model.

## 5. Conclusions

This paper scientifically validates this recommendation from medical practice and draws the following conclusions:

- If the bone is healthy with a good mineral density, so it has good physical properties (for example in the case of secondary arthrosis), then it is not necessary to use the tibial extension in the primary TKA surgery, because the benefits it brings are fewer than the long-term negative consequences.
- If the properties of the bone are altered, then it is necessary to use the tibial extension in the primary TKA operation, because it increases the life of the knee implant.

In the future, we are planning to extend the research to different sizes of tibial stems and analyze their behavior in the same circumstances used in this study, and we will try to define an applicable guideline in terms of picking the optimal stem size depending on the degree of structural bony damage.

## Figures and Tables

**Table 1:** Bone properties of virtual model.

	Density	Young’s Modulus (MPa)	Poisson’s Ratio
	(kg/m^3^)		υ
Cortical bone	1800	18,000	0.3
Healthy cancellous bone	600	700	0.2
Unhealthy cancellous bone	1000	400	0.2

**Table 2:** Implant properties of virtual model.

	Density	Young’s Modulus (MPa)	Poisson’s Ratio
	(kg/m^3^)		υ
Cement	1100	2150	0.48
Polyethylene insert	952	1070	0.41
Tibial component	4428	104,800	0.31
Tibial extension	4428	104,800	0.31

**Table 3:** Stress and displacement values in the static compression regime.

**Healthy Bone**	**Max Stress (MPa)**	**Max Displacement (mm)**
Without tibial stem	18.04	0.1148
With tibial stem	21.2	0.1192
**Unhealthy Bone**	**Stress Max (MPa)**	**Displacement Max (mm)**
Without tibial stem	15.85	0.1047
With tibial stem	6.69	0.0645

**Table 4:** Number of load cycles to failure.

	Healthy Bone		Unhealthy Bone	
	1	2	1	2
Without tibial stem	1,668,583	2,501,725	1,668,583	2,501,725
With tibial stem	6,667,433	7,668,583	4,168,008	5,834,292

**Table 5:** Stress and displacement in linear compression dynamic regime.

	Tensile Stress			General Displacement			*Z*-Axis Displacement		
	Max (MPa)	1 (MPa)	2 (MPa)	Max (mm)	1 (mm)	2 (mm)	Max (mm)	1 (mm)	2 (mm)
Healthy bone									
Without tibial stem	21.070	2.700	4.792	0.080	0.007	0.005	0.080	0.006	0.005
With tibial stem	15.010	0.470	2.940	0.081	0.006	0.003	0.079	0.004	0.003
Unhealthy bone									
Without tibial stem	9.707	0.893	0.472	0.080	0.006	0.004	0.079	0.005	0.004
With tibial stem	9.750	0.715	0.407	0.084	0.005	0.003	0.082	0.004	0.002

## Data Availability

Not applicable.
